# Effectiveness of Topical Ganciclovir 2% Monotherapy Versus Combined Steroid Therapy in Cytomegalovirus Endotheliitis

**DOI:** 10.3390/jcm11195811

**Published:** 2022-09-30

**Authors:** Yu-Wei Kuo, En-Che Chang, Chia-Yi Lee, Shwu-Huey Lee, I-Chia Liang, Yi-Chun Chen, Yu-Chih Hou

**Affiliations:** 1 Department of Ophthalmology, Cathay General Hospital, Taipei 106438, Taiwan; 2 Institute of Public Health, Taipei Medical University, Taipei 110301, Taiwan; 3 Institute of Medicine, Chung Shan Medical University, Taichung 402306, Taiwan; 4 Nobel Eye Institute, Taipei 100008, Taiwan; 5 Department of Ophthalmology, Jen-Ai Hospital Dali Branch, Taichung 412224, Taiwan; 6 Department of Ophthalmology, Tri-Service General Hospital, Taipei 114202, Taiwan; 7 Department of Ophthalmology, National Taiwan University Hospital, College of Medicine, National Taiwan University, Taipei 100225, Taiwan; 8 Department of Ophthalmology, College of Medicine, Fu Jen Catholic University, New Taipei City 242062, Taiwan

**Keywords:** cytomegalovirus, corneal endotheliitis, ganciclovir, corticosteroid

## Abstract

We aimed to report the clinical manifestations of cytomegalovirus (CMV) corneal endotheliitis and the results of long-term treatment with topical ganciclovir 2% with and without steroids. This retrospective, interventional study included 15 eyes of 13 patients diagnosed with CMV corneal endotheliitis by positive CMV DNA and treated with long-term topical ganciclovir 2% eye drops at a tertiary referral center and the median follow-up period was 17 months. Ocular manifestations included keratic precipitates (KPs) (100%), elevated IOP (93.3%), iritis (60%), corneal edema (60%), and moth-eaten iris atrophy (60%). After long-term treatment, corneal edema, iritis, and KPs significantly decreased (effect size: 72%, 76% and 70%, respectively; *p* = 0.024, *p* = 0.006 and *p* < 0.001, respectively). Both the logMAR acuity and IOP significantly improved (median logMAR was 0.52 before treatment and 0.22 after treatment; median IOP was 42 mmHg before treatment and 12 mmHg after treatment; *p* = 0.001 and *p* < 0.001, respectively). The ECD was maintained (effect size: 80%), and the percentage of hexagonal cell ratio of endothelial cells significantly improved after treatment (effect size: 82%; *p* = 0.035). Fewer anti-glaucoma medications were used in the non-steroid group (effect size: 79%; *p* = 0.034). Long-term maintenance treatment with topical ganciclovir 2% monotherapy not only provides effective therapy and reduces recurrence, but also decreases the high IOP related to the combination of steroids used.

## 1. Introduction

Cytomegalovirus (CMV) keratitis has been increasingly found in immunocompetent patients over the past decade [1,2]. Since the first case of CMV corneal endotheliitis in which CMV DNA was detected in the aqueous humor was reported in 2006 [3], increased numbers of publications of CMV corneal endotheliitis in Asia have been reported in the recent years [4,5,6,7,8,9,10,11]. The clinical manifestations of CMV corneal endotheliitis include corneal edema, coin-shaped keratic precipitates (KPs), elevated intraocular pressure (IOP), iritis, and endothelial cell loss [5,12,13,14]. Delays in the diagnosis may cause irreversible corneal endothelial decompensation [5], which is vision-threatening. Therefore, proper and early anti-CMV treatments are important.

The mainstay of anti-CMV therapy is ganciclovir and valganciclovir [15]. The treatment regimens include systemic ganciclovir, oral valganciclovir, topical ganciclovir, intravitreal ganciclovir, or a combination [6,8,12,13,15]. Considering the risk of systemic side effects, such as renal failure and bone marrow suppression of systemic ganciclovir [16], long-term maintenance therapy with topical ganciclovir has been suggested to prevent the recurrence of endotheliitis [6]. Recently, topical ganciclovir 2% has been reported to effectively treat and prevent the recurrences of endotheliitis [9,11]. Topical ganciclovir combined with topical corticosteroids as a maintenance treatment has been shown to effectively preserve the endothelial function [9,10]. However, due to the potential risk of steroid-induced glaucoma and viral replication [10,17], the long-term benefit and safety profile between the monotherapy and combined corticosteroids therapy needs to be evaluated.

The purpose of this study was to present the clinical characteristics of CMV corneal endotheliitis and investigate the long-term therapeutic outcomes of topical ganciclovir 2% with and without topical corticosteroids.

## 2. Materials and Methods

### 2.1. Participants

**Figure 1:**
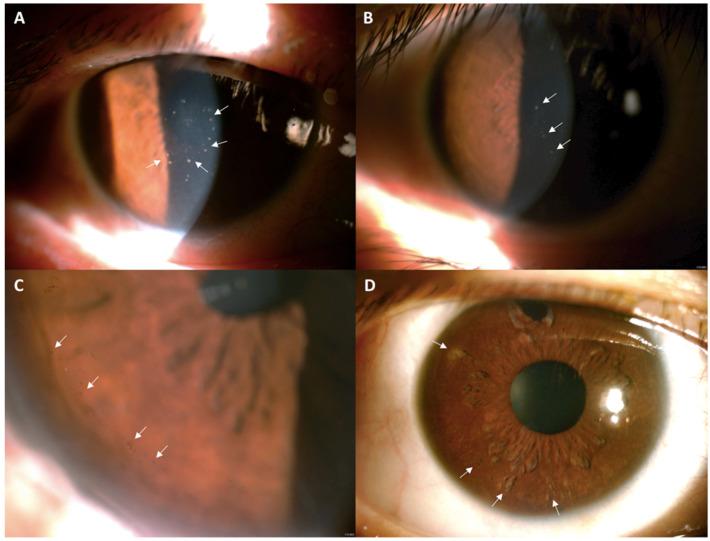
Clinical manifestations of cytomegalovirus corneal endotheliitis by slit-lamp biomicroscopy. (**A**) Typical coin-shaped keratic precipitates (KPs) (white arrows) in the central cornea of case 1. (**B**) Some mutton-fat KPs (white arrows) in case 7. (**C**) Pigmented KPs (white arrows) in the peripheral cornea of case 10. (**D**) Moth-eaten iris atrophy (white arrows) in case 3.

This retrospective study was conducted in all patients with CMV corneal endotheliitis, who received treatment with topical ganciclovir 2% at the Department of Ophthalmology at Cathay General Hospital in Taipei, Taiwan, from March 2017 to February 2021. The patients in this study were diagnosed with CMV endotheliitis based on the clinical manifestations of characteristic KPs, included (1) coin-shaped KPs, (2) pigmented KPs, and (3) mutton-fat KPs, iritis, moth-eaten iris (focal depigmentation and iris atrophy), elevated IOP > 21 mmHg, corneal edema, and qualitative PCR of the aqueous humor (Figure 1). Patients who received prior keratoplasty, including penetrating keratoplasty or Descemet stripping automated endothelial keratoplasty, were excluded because the essential long-term use of corticosteroids for decreasing rejection rates may cause bias.

### 2.2. CMV PCR Test

Qualitative PCR examination using an aqueous sample obtained from each of the 15 eyes (100%) was positive for CMV DNA. An aliquot (0.1 mL of aqueous humor) was tapped from anterior chamber taps for CMV DNA extraction. The two PCR primer sets used for CMV were 5′-CCTAGTGTGGATGACCTACGGGCCA-3′ and 5′-CAGACACAGTGTCCTCCCGCTCCTC-3′ for the product of 250 base pairs, and 5′-AGCTGCATGATGTGAGCAAG-3′ and 5′-GAAGGCTGAGTTCTTGGTAA-3′ for the product of 146 base pairs. Either fragment detected by electrophoresis would indicate a positive result for CMV. Internal controls and negative controls were also used in the CMV PCR test [18,19].

### 2.3. Treatment

After the detection of CMV by PCR, the application of topical ganciclovir 2% every 1 or 2 h was initially performed with optional topical betamethasone 0.1% four times daily, which was described if there was at least 1 grade of the anterior chamber (AC) inflammation for one or two weeks. When corneal edema and KPs gradually improved and responded to the treatment, topical ganciclovir 2% was tapered to four times daily for long-term treatment and topical betamethasone 0.1% was changed to topical fluorometholone 0.1% four times or less daily in most cases. The grading of the corneal edema was scored from 0 to 4; 0 means no edema, 1 indicates slight stromal edema, 2 indicates diffuse stromal edema, 3 indicates diffuse stromal edema with epithelial microcystic edema, and 4 indicates bullous keratopathy [20]. Response to treatment was defined as (1) an improvement of corneal edema with at least one grade decrease, (2) AC inflammation with at least one step decrease, (3) decrease in the number of KPs, and (4) IOP back to the normal range <21 mmHg. Recurrence was defined as one of the following findings during maintenance therapy: (1) flare-up of AC reaction, (2) increased KPs, (3) elevated IOP, and (4) localized corneal edema. We chose clinical diagnosis instead of aqueous PCR to confirm the recurrence because the procedure of aqueous tap itself may cause endothelium damage and the PCR test may reveal false-negative results even when the patients presented with high IOP and increased KPs. All recurrent cases were re-started using the initial dose of topical ganciclovir 2% every 2 h and topical betamethasone 0.1% four times a day.

### 2.4. Outcome Measurement

The chart records of corneal edema, types of KPs, iritis, iris atrophy, best-corrected visual acuity (BCVA), and IOP before and after at least 2 weeks of treatment with topical ganciclovir 2% were reviewed. IOP was measured with pneumotonometer for at least 3 times for each visit. Endothelial cell density (ECD), coefficient of variation (CV), and hexagonal cell ratio (HEX) were examined by specular microscopy (Konan, Japan), and the central ECD was automatically calculated. The medications of anti-glaucoma agents and adjunctive steroids were also reviewed during the follow-up period. The side effects of long-term treatment were also evaluated through subjective complaints and biomicroscopic examination of the ocular surface. The patients with long-term continuous use of betamethasone or fluorometholone over 6 months were defined as the steroid group.

### 2.5. Statistical Analysis

Statistical analysis was performed using the Statistical Package for the Social Sciences software version 20 (SPSS Inc., IBM Company, Chicago, IL, USA). Data are presented as medians with interquartile ranges (IQR). The association between pre-treatment and post-treatment data was analyzed using the Wilcoxon signed-rank test and Fisher’s exact test. Subgroup analysis including the steroid and non-steroid groups, and the recurrence and non-recurrence groups, were analyzed using the Mann–Whitney U test or Fisher’s exact test. Statistical significance was set at *p* < 0.05. Subgroup analysis comparing 3 types of typical KPs and the outcome parameters was analyzed using Kruskal–Wallis test.

## 3. Results

A total of 15 eyes from 13 patients diagnosed with CMV corneal endotheliitis were enrolled. Two patients had bilateral eye involvement. The median age of the 13 patients was 62 years (IQR, 58–68 years). Nine cases (60%) were men and six cases (40%) were women. The median follow-up period was 17 months (IQR, 23–42.5 months). At the initial presentation, typical coin-shaped KPs were observed in nine eyes (60%), pigmented KPs in four eyes (26.6%), and mutton-fat KPs in two eyes (13.3%). Corneal edema was present in 9 eyes (60%), and AC inflammation was observed in nine eyes (60%). Moth-eaten iris atrophy was detected in nine eyes (60%). Elevated IOP was observed in 14 eyes (93.3%), and the median IOP (IQR) was 42 (28–48) mmHg. Anti-glaucoma medications at the time of diagnosis of CMV endotheliitis were received by 13 eyes (86.7%).

**Figure 2:**
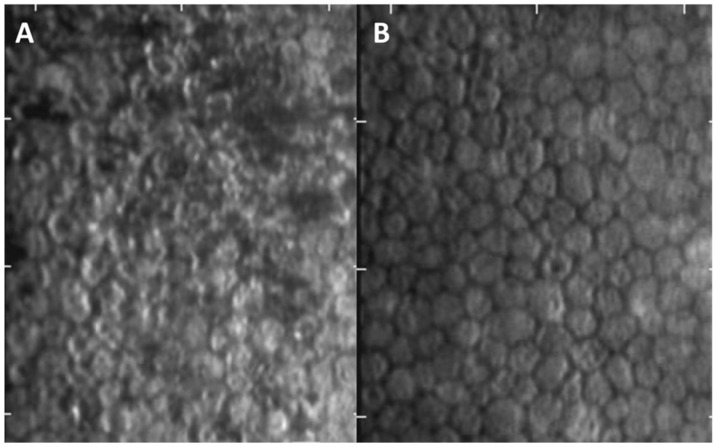
Specular microscopy of case 7. (**A**) Before treatment, the corneal endothelium showed numerous dark cells as pseudoguttata. The endothelial cell density (ECD) was 2331 cell/mm^2^, with marked pleomorphism and polymegathism. (**B**) After ganciclovir treatment, the ECD increased to 2500 cell/mm^2^, and the coefficient of variation value and hexagonal ratio improved from 51% to 43% and 33% to 43%, respectively.

The median logMAR acuity (IQR) improved significantly from 0.52 (0.30–0.52) before treatment to 0.22 (0.00–0.52) after treatment (*p* = 0.001) (Table 1). The median IOP (IQR) also significantly decreased from 42 (28–48) mmHg to 12 (10–16) mmHg (*p* < 0.001). Thirteen eyes (86.7%) retained corneal clarity (effect size: 72%; *p* = 0.024). Fifteen eyes (100%) presented with different types of KPs, nine eyes (60%) showed complete remission of KPs, while the other six eyes (40%) achieved improvement (effect size: 70%; *p* < 0.001). Among these six eyes with residual KPs, five eyes exhibited typical coin-shaped KPs at the initial presentation. The final median ECD, CV, and final central corneal thickness (CCT) were not significantly different after treatment. The final HEX percentage significantly improved from 33 (27–49) to 38 (34–50) (effect size: 82%; *p* = 0.035) (Figure 2). After the treatment, the number of anti-glaucoma medications required for IOP control significantly decreased (effect size: 79%; *p* = 0.039). Outcome comparison between three types of typical KPs showed no significant difference (all *p* > 0.05).

**Figure 3:**
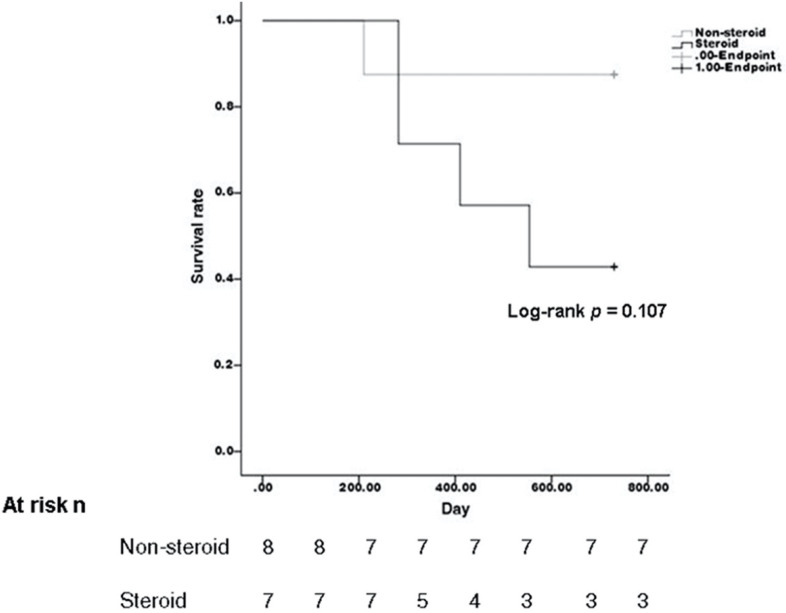
Kaplan-Meier Method comparing the recurrence rate of steroid and non-steroid group.

Compared with the steroid group (seven eyes) and non-steroid group (eight eyes), all of the initial clinical manifestation and most of the outcome parameters were not significantly different (Table 2 and Table 3). The recurrence rate calculated using the Kaplan–Meier method was also not significantly different (*p* = 0.107) (Figure 3). All of the patients presented with improved iritis or no iritis at the end of this study. However, in the steroid group, there were four eyes with iritis (*p* = 0.044). A reduced need for anti-glaucoma medications was observed in both groups after treatment and was significantly lower in the non-steroid group (*p* = 0.034).

During the follow-up period, five eyes (33.3%) in three patients with disease recurrence were detected under maintenance therapy with topical 2% ganciclovir. After our treatment, the clinical signs resolved within 1–2 weeks. Among these five recurrent eyes, three eyes developed mild AC inflammation with typical coin-shaped KPs with or without increased IOP, one eye developed increased IOP with mutton-fat KPs, and one eye developed pigmented KPs only. Among these five recurrent cases, four eyes received long-term topical betamethasone 0.1%.

## 4. Discussion

The clinical features of CMV corneal endotheliitis may present as other herpes viral infections, Posner-Schlossman syndrome, Fuchs’ heterochromic iridocyclitis, or iridocorneal endothelial (ICE) syndrome [3,5,12,14,21]. Coin-shaped KPs and raised IOP are the characteristic findings of CMV corneal endotheliitis [13,14]. Other presentations included pigmented KPs, non-pigmented fine KPs, corneal edema, iritis, and moth-eaten iris atrophy [6,9,10,12,21]. According to a large retrospective study of 106 eyes with CMV endotheliitis [5], the most common features are typical coin-shaped KPs (70.6%) and corneal edema (73.4%). In our study, the five most common features were coin-shaped KPs (60%), iritis (60%), moth-eaten iris (60%), corneal edema (60%), and elevated IOP (93.3%). In addition to the presence of typical coin-shaped KPs, other types of KPs, including pigmented or mutton-fat KPs, should also be considered as one of the clinical clues for CMV corneal endotheliitis. In addition, the moth-eaten iris was characteristic of CMV endotheliitis, which was not present in other herpetic keratitis or ICE syndrome. In clinical practice, if patients present with more than one of five features, especially the coin-shaped KPs and elevated IOP, the diagnosis of CMV corneal endotheliitis should be taken into consideration and can be confirmed by aqueous tapes for CMV PCR.

The mainstay of therapy is ganciclovir and valganciclovir, which can inhibit DNA polymerase and its synthesis [14]. Several treatment regimens have been used in clinical practices, including systemic and topical use [6,8,12,13,15]. Considering the potential adverse effects of systemic ganciclovir [16], topical ganciclovir is an alternative that can reach therapeutic levels in the cornea and aqueous humor and is significantly safer for long-term treatment [5,22,23]. Different concentrations of topical ganciclovir, including 0.15% gel and 0.5%–2% eye drops, have been reported to treat CMV corneal endotheliitis with some success [2,6,10,11]. According to previous clinical studies, ganciclovir 2% seemed able to achieve an inhibitory concentration for CMV in aqueous humor and effectively clear the viral load [6].

In our study, the majority of the eyes retained their corneal clarity (86.7%) and achieved complete remission of KPs (60%) after long-term topical ganciclovir 2% treatment. There were no significant side effects, such as corneal epithelial damage or conjunctival injection. The final median ECD did not decrease compared to the baseline density, and the final median percentage of HEX was significantly improved, indicating that topical ganciclovir 2% can not only preserve the endothelial cell count, but also help to improve the function of endothelial cells, contributing to the recovery of corneal edema. In patient 7, corneal edema and high IOP were noted before diagnosis. Trabeculectomy was performed due to persistently high IOP, even when topical ganciclovir 2% was prescribed. During the operation, a repeated aqueous tap for CMV PCR showed a negative result, which indicated the effective efficacy of topical ganciclovir 2% treatment in clearing the CMV viral load. However, a delayed diagnosis and treatment in our patient demonstrated irreversible damage to both the trabecular meshwork and the endothelial cells, even when the CMV was cleared.

According to previous studies, the combination treatment of topical corticosteroid and ganciclovir showed some favorable results for the maintenance of corneal clarity and prevention of recurrence [8,9,10]. However, the long-term use of corticosteroids may cause steroid-induced glaucoma and accelerate cataract progression in phakic eyes [9,10]. Although fluorometholone is considered to have minimal ocular penetration and minimal risk of steroid-induced glaucoma, the incidence rate of fluorometholone-induced IOP elevation was reported to range from 2% to 13% [24,25,26,27]. According to our results, topical ganciclovir 2% without the long-term use of steroids showed no significant difference in most of the outcome parameters, except for iritis and the demand for anti-glaucoma medication. There were four eyes with iritis in the steroid group, which may be related to the preference for steroid use in more severe iritis in clinical practice. The requirement for anti-glaucoma medication was significantly lower in the non-steroid group, which implies better control of IOP without the use of corticosteroids. In addition to the better control of IOP, the reduced use of anti-glaucoma medications also takes advantage of reducing ocular surface damage [28]. The use of corticosteroids may increase the risk of viral replication [17] and CMV trabeculitis. Therefore, it was rational to use topical ganciclovir 2% alone as long-term maintenance treatment without steroids, which also achieves better IOP control.

In our study, five eyes (33.3%) with disease recurrence were noted. Studies regarding the long-term maintenance therapy of topical ganciclovir for CMV corneal endotheliitis are scarce [6,9,10,11]. There is a consensus that recurrence may occur after the discontinuation of ganciclovir [10,29,30,31], which emphasizes the importance of long-term maintenance treatment. However, despite the continued use of topical anti-CMV medications, 7.35% to 57.14% of the eyes still develop recurrence, and a lower concentration of topical ganciclovir may contribute to the higher recurrence rate [9,10,11,31]. Despite the lower concentration of topical ganciclovir, the presence of resistant CMV strands may lead to recurrence, too. These patients may need a more frequent dose of topical ganciclovir to suppress the re-activation. The different definition standards of recurrence in these studies may also explain the wide variety of recurrence rate. The relatively high recurrence rates in our study may be due to the strict definition of recurrence as any presentation with one of the recurrent signs counted. Among these five recurrent eyes, four eyes received continuous steroid use. Although the use of steroids was not statistically different between the recurrence and non-recurrence groups, the percentage of the use of steroids was higher in the steroid group. Further study with a larger sample size may be needed.

The limitations of our study included the retrospective data collection, heterogeneity of follow-up duration, small sample size, and non-single doctors. In addition, the PCR testing in our laboratory was only available for CMV, but not for HSV or VZV. According to previous reports [5,13,32], the incidence of viral endotheliitis diagnosed with more than two herpes viruses simultaneously was very rare. Thus, we treated all of our patients with topical ganciclovir 2% after the detection of CMV by PCR, and their clinical presentation was significantly improved after the treatment. Although the sample size in our study was relatively small, the number of cases (15 eyes) was larger than the other case series mentioned for the long-term maintenance treatment with topical ganciclovir [6,9,10,13].

## 5. Conclusions

In conclusion, long-term maintenance treatment with topical ganciclovir 2% could decrease the IOP, improve VA, restore corneal endothelium function, and reduce the recurrence. Topical ganciclovir 2% monotherapy may be good enough for long-term maintenance treatment, while long-term steroids may increase the demand for anti-glaucoma medication. CMV corneal endotheliitis was characterized by high IOP, coin-shaped KPs, and moth-eaten iris atrophy. With more than one of the above characteristic features presented, CMV corneal endotheliitis should be highly suspected and confirmed by PCR. A further large-scale, extended follow-up prospective study may be warranted to evaluate the efficacy of long-term topical ganciclovir.

## Figures and Tables

**Table 1:** The Ophthalmic Parameters Before and After Treatment in Cytomegalovirus Corneal Endotheliitis.

Parameter	Pre-Treatment (*n* = 15)	Post-Treatment (*n* = 15)	*p*-Value
IOP (mmHg, median (IQR))	42.00 (28.00–48.00)	12.00 (10.00–16.00)	<0.001 *
BCVA (LogMAR, median (IQR))	0.52 (0.30–0.52)	0.22 (0.00–0.52)	0.001 *
Corneal edema grading			0.024 *
0	6	13	
1	8	2	
2	1	0	
KP	15	6	<0.001 *
Iritis 0 Trace 1+ ≥2+	6 3 5 1	11 3 1 0	0.006 *
ECD (mm^2^, median (IQR))	1976 (1647–2331)	1900 (1600–2500)	0.302
CV (%, median (IQR))	49 (42–54)	45 (41–67)	0.712
HEX (%, median (IQR))	33 (27–49)	38 (34–50)	0.035 *
CCT (mm, median (IQR))	597 (571–629)	597 (563–632)	0.607
Number of anti-glaucoma drug 0 1 2 3 4	2 2 5 4 2	5 7 3 0 0	0.039 *

* *p* < 0.05. *n* = number; IQR = interquartile range; BCVA = best-corrected visual acuity; IOP = intraocular pressure; KP = keratic precipitates; ECD = endothelial cell density; CV = coefficient of variant; HEX = hexagonal ratio; CCT = central corneal thickness.

**Table 2:** The Difference of Initial Clinical Manifestation Between the Steroid Subgroup and Non-steroid Subgroup.

Clinical Manifestation	Steroid Group (*n* = 7)	Non-Steroid Group (*n* = 8)	*p*-Value
IOP (mmHg, median (IQR))	29.00 (25.00–51.00)	44.50 (33.00–48.00)	0.245
BCVA (LogMAR, median (IQR))	0.52 (0.30–0.52)	0.46 (0.13–0.88)	0.632
Corneal edema grading 0 1	3 4	3 4	0.626
2	0	1	
KP	7	8	1.000
Iritis 0 Trace 1+	1 2 3	5 1 2	0.246
3+	1	0	
ECD (mm^2^, median (IQR))	1969 (1621–2233)	1976 (1647–2558)	0.685
CV (%, median (IQR))	47 (42–59)	49 (41–54)	0.870
HEX (%, median (IQR))	34 (29–41)	33 (27–50)	0.871
CCT (mm, median (IQR))	615 (597–629)	591 (569–622)	0.364
Number of anti-glaucoma drug 0 1 2	1 1 3	1 1 2	0.710
3	2	2	
4	0	2	

*n* = number; IQR = interquartile range; BCVA = best-corrected visual acuity; IOP = intraocular pressure; KP = keratic precipitates; ECD = endothelial cell density; CV = coefficient of variant; HEX = hexagonal ratio; CCT = central corneal thickness.

**Table 3:** The Difference of Post-treatment Outcome Between the Steroid Subgroup and Non-steroid Subgroup.

Outcome	Steroid Group (*n* = 7)	Non-Steroid Group (*n* = 8)	*p*-Value
IOP (mmHg, median (IQR))	12.00 (10.00–17.00)	12.00 (9.00–15.75)	0.613
BCVA (LogMAR, median (IQR))	0.15 (0.00–0.52)	0.26 (0.02–0.62)	0.867
Corneal edema grading 0 1	7 0	6 2	0.467
KP	3	3	0.622
Iritis 0 Trace 1+	3 3 1	8 0 0	0.044 *
ECD (mm^2^, median (IQR))	1900 (1669–2747)	1658 (1540–2464)	0.281
CV (%, median (IQR))	54 (45–58)	43 (41–69)	0.536
HEX (%, median (IQR))	38 (37–60)	39 (34–45)	0.336
CCT (mm, median (IQR))	610 (535–642)	586 (566–599)	0.397
Number of anti-glaucoma drug 0 1 2	3 1 3	2 6 0	0.034 *
Recurrence	4	1	0.100
Duration of GCV use (month, median (IQR))	24.5 (13.5–40)	11 (9–13.5)	0.002 *
Duration of steroid use (month, median (IQR))	19.5 (13.5–30)	0.5 (0–1)	<0.001 *

* *p* < 0.05. *n* = number; IQR = interquartile range; BCVA = best-corrected visual acuity; IOP = intraocular pressure; KP = keratic precipitates; ECD = endothelial cell density; CV = coefficient of variant; HEX = hexagonal ratio; CCT = central corneal thickness; GCV = ganciclovir.

## Data Availability

The data from this study are available from the corresponding author upon reasonable request.
